# Avian vision models and field experiments determine the survival value of peppered moth camouflage

**DOI:** 10.1038/s42003-018-0126-3

**Published:** 2018-08-17

**Authors:** Olivia C. Walton, Martin Stevens

**Affiliations:** Centre for Ecology & Conservation, College of Life & Environmental Sciences, University of Exeter, Penryn Campus, Penryn, Cornwall, TR10 9FE UK

## Abstract

Animal defensive coloration has long provided many important examples of evolution and adaptation. Of these, industrial melanism in the peppered moth is the classic textbook example of evolution in action, whereby dark and pale morphs suffer differential predation in polluted and unpolluted woodland based on their camouflage. Despite extensive work, a striking gap remains in that no study has ever objectively quantified their camouflage or related this directly to predation risk. Here we use image analysis and avian vision models to show that pale individuals more closely match lichen backgrounds than dark morphs. Artificial predation experiments in unpolluted woodland show 21% higher survival rates of pale than melanic individuals. Overall, we provide the strongest direct evidence to date that peppered moth morph frequencies stem from differential camouflage and avian predation, providing key support for this iconic example of natural selection.

## Abstract (web summary)

Olivia Walton and Martin Stevens revisit the classic example of the peppered moth, objectively quantifying moth camouflage and predation risk. With bird vision models, pale individuals more closely match lichen backgrounds, and survive better, providing support for this iconic example of natural selection.

## Introduction

Across nature, many taxa have evolved camouflage to avoid detection or recognition^1^. Animal defensive coloration has long provided flagship examples to illustrate and defend evolution and adaptation, including early work by Bates and Wallace on mimicry and camouflage^2,3^. Arguably, the most important example of evolution in action is that of industrial melanism and the peppered moth (*Biston betularia*). During the Industrial Revolution (*ca*. 1760–1840), there was a rise of a new dark melanic (*carbonaria*) form in conjunction with a corresponding decline of the pale (*typica*) morph^4^. The former is widely thought to have been well camouflaged against birds on trees where atmospheric pollution had killed off the lichen and soot particulates coated the bark, with *typica* effectively hidden on lichen-covered trunks and branches in unpolluted woodland. This was supported by the classic work of Kettlewell^5,6^, who demonstrated strong selection against *typica* in polluted woodland and against *carbonaria* in unpolluted woodland where lichen persisted. Further work around this time^7,8^ supported Kettlewell and found that, despite persisting at frequencies as high as 80%, *carbonaria* still incurred a selective disadvantage in unpolluted areas. The introduction of the Clean Air Acts (1950s) provided further support^9^, whereby pollution declined, lichen populations recovered, and there has since been a rise in the frequency of *typica* and a decline in *carbonaria*^10^. Correspondingly, recent work has shown strong selection pressure against the melanic form, driven by differential avian predation^11^. Therefore, this example provides key evidence for natural selection, shows the importance of a greater understanding of the ways in which anthropogenic activity influences defensive adaptations, and can provide further insight into both predator-prey dynamics and other anthropogenic impacts (e.g. climate change)^12^.

Despite the above, and considerable supporting work, this example of natural selection has been repeatedly attacked through unsupported claims of fraudulence on Kettlewell’s behalf^13^. These claims have been firmly rebutted^14,15^, but more importantly, the publication of scientific criticisms^4,16,17^ ultimately led to some doubts in the scientific community and furthered the promotion of an anti-evolution agenda from the non-scientific community^18^. Most of these criticisms and uncertainties have since been largely addressed (for example, natural resting sites), and the validity of the original studies confirmed through further experiments providing reliable evidence indicating bird predation is the most important selective factor driving camouflage in *Biston betularia*^11^. However, there remains a crucial gap in this example—remarkably, no study has quantified the camouflage of peppered moths, or related this to survival against predators in controlled experiments. This is crucial because humans and birds have visual systems differing in terms of number of receptor types, receptor sensitivity, and the ability of birds to perceive ultraviolet (UV) light^19^. With *typica* appearing speckled under UV light (due to white wing scales strongly reflecting and black scales absorbing UV^20^) and crustose lichen species on which *typica* rest^21^ reflecting similar UV patterns, such natural backgrounds may consequently better conceal *typica* from its avian predators. In addition, for camouflage to work an object must closely resemble its background, and a fundamental criterion of camouflage theory is that the closer an object matches the background the less likely it is to be seen^1,22^. To date, evidence that peppered moths are truly camouflaged has been indirect or subjective, being based on human assessment of either the moths directly or images of them.

Using museum specimens, including some of Kettlewell’s original collections, we used digital image analysis^23^ and models of avian vision^24^ to quantify the camouflage match for colour and luminance (lightness) of *typica* and *carbonaria* forms against lichen and plain tree bark (see Methods). We expected that *typica* would share greater similarities with crustose lichen backgrounds in comparison to plain bark backgrounds, whereas the reverse should be seen with *carbonaria*. Comparisons of 65 *typica* and 65 *carbonaria* individuals, each against a different sample of lichen and bark, allowed us to calculate discrimination values (just noticeable differences; JNDs) for colour and luminance of each moth to each background. Increasingly higher JND values indicate greater mismatch, and values close to 1.00 suggest camouflage so effective that colours cannot be distinguished between moths and their resting background.

Next, we performed predation experiments^25,26^ in unpolluted woodland with substantial lichen density (mostly in Cornwall, UK) to compare the likelihood of detection of *typica* and *carbonaria* morphs by avian predators. A widely used and powerful technique is to use artificial prey items designed to resemble real animals to predator vision^25^ (see “Methods” section); effective for monitoring survival over time^26^ when presented with an edible component and pinned to natural backgrounds in the field. We created artificial moths matching the appearance of *typica* and *carbonaria* forms using images of peppered moth museum specimens and measured predation over time for each morph, predicting that survival would be higher of models matching the *typica* morph. Overall, we show that, as predicted, to avian vision *typica* individuals of the peppered moth more closely match lichen covered bark, whereas *carbonaria* individuals more closely match plain bark. Furthermore, these differences translate into a strong survival advantage of *typica* individuals in unpolluted woodland.

## Results

### Matches of morphs to different resting backgrounds

**Fig. 1:**
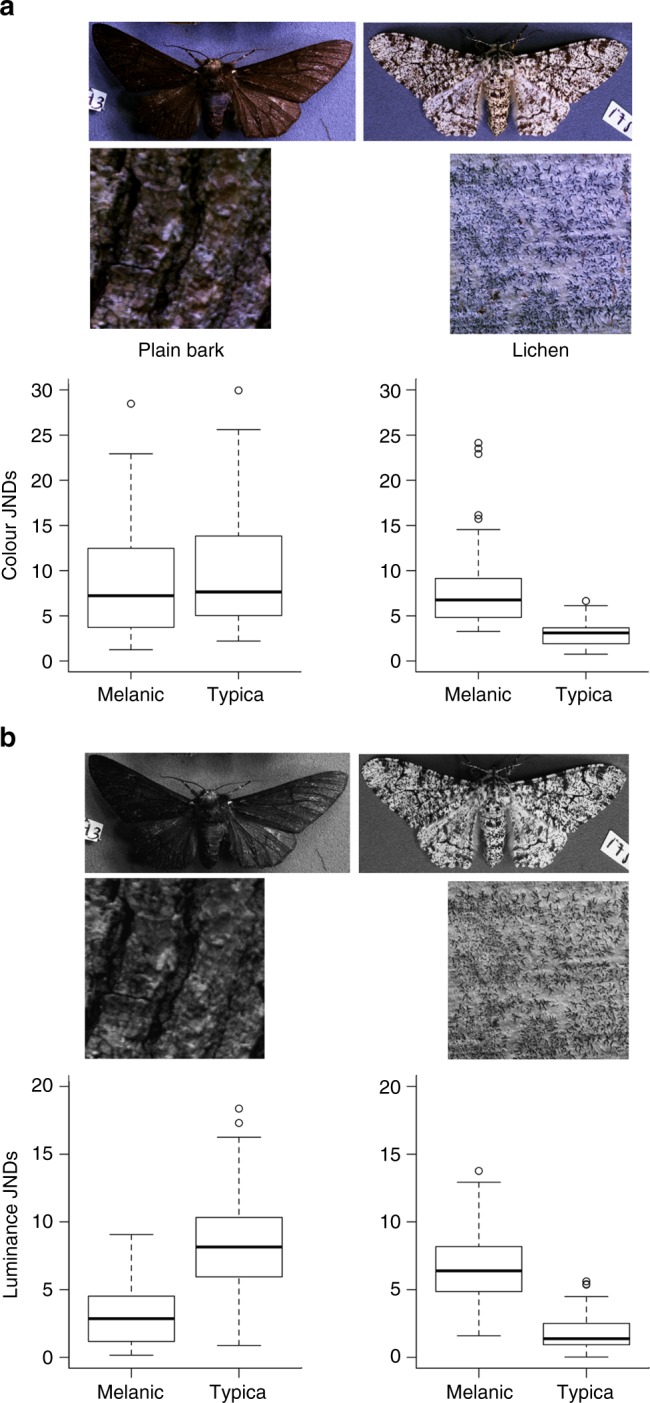
Camouflage of peppered moth morphs to avian vision. Images show a melanic and a typical peppered moth morph to avian vision, along with samples of plain bark and lichen. **a** This set of images represent colour data (*n* = 130), comprised of cone response data for a longwave, mediumwave, shortwave, and UV cones (with UV and shortwave data combined into the blue image channel as images have only three layers). **b** This set of images represent data from avian double cones, showing luminance (*n* = 130). These images illustrate the better match for colour and luminance of *typica* compared to carbonaria against lichen backgrounds. Graphs are just noticeable difference (JND) results for colour (**a**) and luminance (**b**) of *typica* and *carbonaria* specimens against plain bark and lichen. JND data was statistically analysed using a general linear model, with colour data log-transformed. For colour (**a**) between the morphs, plain bark did not display significance (*p* = 0.19) whereas lichen bark did (*p* = 6.66e^−14^). Both morphs displayed statistical significance for luminance (**b**); typica (*p* < 2e^−16^) and melanic (*p* < 2e^−16^). Boxplots display untransformed average JND values (bold line), the interquartile range (box component), range of minimum and maximum JND values (horizontal lines either end of range), and circle symbols signifying outlier results

Peppered moth morphs differed significantly against lichen bark backgrounds (*F*_1,129_ = 129.99, *n* = 130, *p* = 6.66e^−14^, Fig. 1), whereby *typica* morphs displayed low chromatic differences, or good camouflage (mean JND = 2.99 ± 1.17 standard error), and *carbonaria* had a poorer match to the background (8.03 ± 0.83 JND). There was no significant difference in the colour match between the morphs on plain bark backgrounds (*F*_1,129_ = 1.66, *n* = 130, *p* = 0.19); *typica* (9.81 ± 2.17 JND) and *carbonaria* (8.95 ± 1.53 JND). For luminance, there was also a significant difference in matching between morphs to lichen (*F*_1,129_ = 196.9, *n* = 130, *p* < 2e^−16^), with *typica* (1.82 ± 0.67 JND) predicted to be close to indistinguishable in luminance to lichen, compared to *carbonaria* (6.64 ± 0.48 JND) that has greater achromatic contrast. Furthermore, there was a significant difference in luminance camouflage between morphs on plain bark (*F*_1,129_ = 108.93, *n* = 130, *p* < 2e^−16^), with *typica* showing greater difference and worse camouflage (8.56 ± 1.03 JND) in comparison to *carbonaria* (3.07 ± 0.73 JND), which showed a close match. Overall, our results support the expectations that *typica* is a much closer match to crustose lichen, whereas *carbonaria* a closer match to plain bark (Fig. 1).

### Survival of morphs in unpolluted woodland

**Fig. 2:**
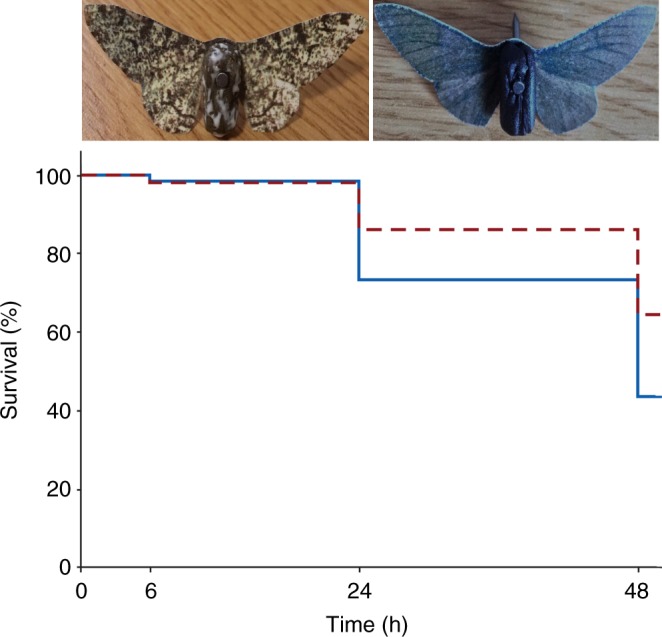
Visualisation of the artificial predation experiment. Examples of the artificial moth targets made to match *typica* and *carbonaria* specimens show the components of the pastry body and the digitally colour calibrated paper wings. Statistical analysis was conducted to produce the non-parametric distribution plot of survival over time, using Kaplan–Meier estimation. Higher survival of targets matching *typica* moths than *carbonaria* moths were seen; graphically represented by the red dashed and solid blue lines, respectively (*n* = 500; *p* < 2e^−16^)

**Fig. 3:**
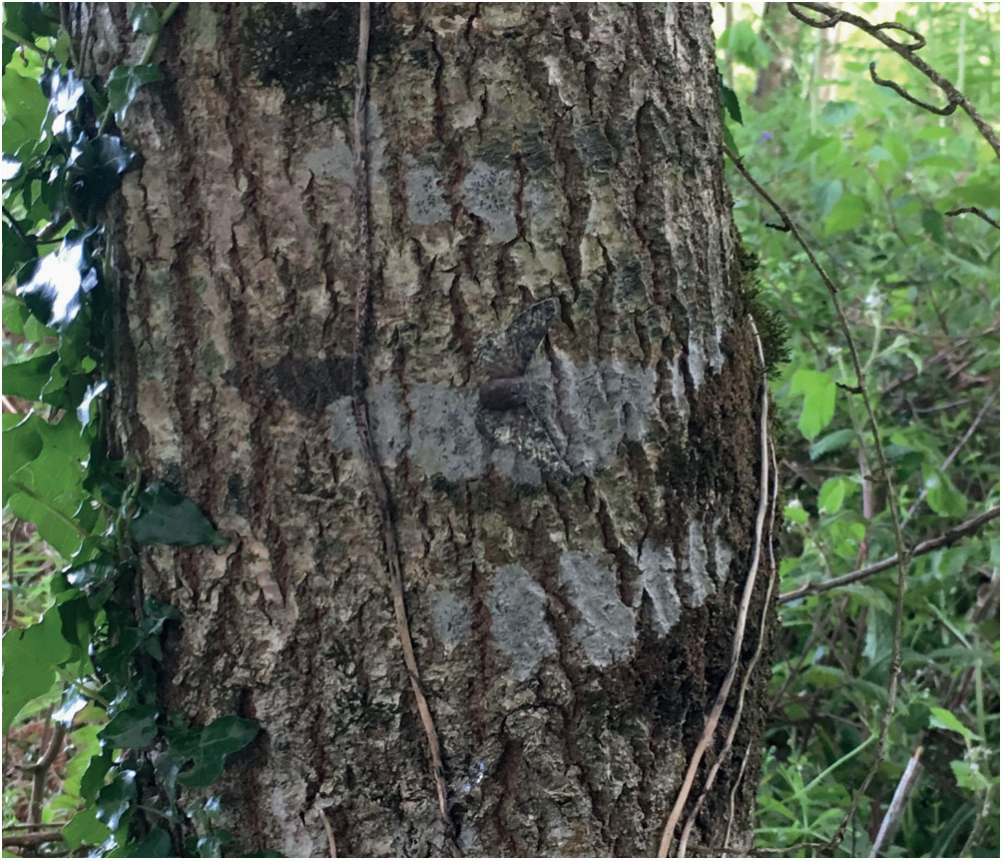
A target matching a typica moth pinned to a lichen covered tree in woodland in the predation experiments

We next performed predation experiments^25,26^ in unpolluted woodland. Artificial moths matching the appearance of *typica* and *carbonaria* forms with an edible pastry body^27^ were pinned to lichen covered tree trunks (confirmed as key resting sites for both morphs^4^; Figs 2 & 3) in a randomised block design in different woodland areas^26^. We expected that there would be a higher survival of *typica* than *carbonaria* targets. Survival was measured over a 48-h period across ten experimental blocks and significantly differed between *typica* and *carbonaria* (*X*^2^ = 22.23, d*f* = 1, *n* = 500, *p* < 2e^−16^; Fig. 2). Survival probability estimates at 48 h were 0.43 ± 0.03 for *carbonaria* and 0.64 ± 0.03 for *typica* (Figs 2, 3). No morph-related censoring bias was observed for non-avian predation or disappearing targets: 20 *typica* and 17 *carbonaria* treatments required censoring. These results, overall, indicate that *typica* had an ~21% greater survival rate than *carbonaria* across the entire experiment.

## Discussion

We have shown that the pale speckled form (*typica*) of the peppered moth has a close match to lichen covered bark for both colour and lightness, whereas the melanic (*carbonaria*) form is poorly matched and closer in lightness to plain bark. Therefore, to avian vision, the *typica* form is indeed better hidden against lichen covered trees than *carbonaria*. Crucially, this translates into a strong survival advantage, with replica models of *typica* being much less likely to be discovered by wild birds when on lichen covered backgrounds. These two components provide substantial direct support for the role of camouflage and differential avian predation in driving the rises and falls of polymorphic frequencies, and specifically the documented increases in *typica* during the post-industrial era^9,10^.

In our assessment of camouflage, the match of *typica* to lichen was very close using a model of avian vision, with values around 2–3 JNDs. Instead, *carbonaria* was a close match to plain bark for luminance, although not colour. In contrast, all moths had JNDs of around 7–9 for both colour and luminance against their incorrect background, indicating a substantially poorer match and diminished camouflage. These data are already in very close accordance with our predictions, but in addition, we used museum specimens and the resulting match between fresh specimens resting on in situ lichen under natural lighting may be closer. We also took average matches over the entirety of the moth wing patterns, which may produce a lower match (and a more conservative camouflage estimate) than between specific patches of colour on the moths to the background. In addition to the above considerations, it should be noted here that we have not tested how closely *carbonaria* would resemble trees covered with a fine layer of soot; most likely, the match would be even closer. However, conducting such an experiment would present two key issues that are hard to resolve. First, it is unclear as to how much soot should be applied to the bark samples because too little or too much may skew the results substantially. One would have to rely on historical accounts of how much soot was around in polluted woodland, and this would not necessarily have been accurately recorded or replicable. Second, as the soot during the Industrial Revolution was deposited from the settling of particulates from the atmosphere, it would be difficult to correctly replicate this natural scenario through artificial application. Liebert and Brakefield^28^ noted that Kettlewell documented soot particulates being built up on trees based upon patterns of drainage, and so an effective replication of this would need information about the type of drainage pattern on the trees involved in Kettlewell’s and others’ experiments, for which we are not aware of any information. Finally, the plain bark we use here is an appropriate comparison in many regards, since in many polluted areas it is not necessarily the presence of soot that matters, but pollution killing off lichen rendering the tree bark bare.

Although the colour match of our artificial prey pastry bodies to the target wings and relevant natural background was not close for the *carbonaria* models compared to the *typical* ones (see “Methods” section), this is highly unlikely to explain our results and detection should primarily be based on the wing coloration, for several reasons. First, as discussed elsewhere^29^, the overall area of the wings is many times greater than that of the body (here, seven times larger: 442 mm^2^ for wings vs. 60 mm^2^ for the body), and a key predictor of detection is object size. In addition, the prey bodies only touch the background bark at the top and bottom of the targets, and so this small area of contrast with the bark background is unlikely to be important (though body-wing contrast may have more influence). Second, numerous past experiments have investigated the effect of an edible body vs. target appearance on detection / predation, and repeatedly shown no or minimal effect of body presence or appearance. These experiments have, for example, compared detection and attack rates on targets with camouflage or eyespot markings against birds, whereby an edible body (a dead mealworm larvae or pastry body, often mismatching the main target) was placed either directly on top of the wings or underneath, partly projecting out^26,29–34^. In addition, studies have compared results of computer experiments with human subjects searching for computer-generated prey lacking edible bodies with work using the same stimuli types in field experiments^26,34–36^. In all these studies, results are entirely consistent regardless of the placement and presence/absence of a body component. Finally, the detection of small targets and of texture is mediated primarily by luminance rather than colour contrast^37,38^, and in this regard for both morphs the bodies were a close and similar match to the wings and natural background. Therefore, we are highly confident that our results are fully or largely explained by model wing appearance against the background.

Kettlewell^39^ showed that morphs of the peppered moth choose backgrounds to rest on that more closely resemble their appearance (e.g. *carbonaria* on black stripes, *typica* on white stripes). This was also confirmed in later work, although it is not straightforward as there can be variation among individuals, especially melanic ones, and the mechanisms underlying choices are unresolved^40^. Work on other species has shown that individual moths have a remarkable ability to adjust their resting orientation and location to improve their own individual-specific, rather than species- or morph-specific, match to the background^41^. Such behavioural approaches to facilitate camouflage have also been demonstrated in birds and lizards, among other taxa^40,42–44^. Consequently, real live peppered moths may be able to further improve their match to appropriate backgrounds through behavioural responses to ultimately confer a greater survival advantage. While recent work has largely resolved the issue of where these moths rest (lower branch surfaces and trunks^11^), more work is also needed on the fine-scale background resting locations of each morph, including with regards to lichen types. Current work, as here, has focused on crustose lichens rather than foliose species^21,45^. This is partly because the use of crustose lichen is consistent with information on chosen resting positions of moths, and because crustose lichen has been shown to reflect UV in a similar manner to *typica* moths, in comparison to foliose lichen, which absorbs UV creating a poor match with *typica*^4,21^.

While industrial melanism provides an important example of evolution, it is also an early demonstration of how anthropogenic changes affect species and their interactions with their environment. More recent work has investigated if and how camouflaged animals will be affected by climate change^46^, with coral bleaching through ocean acidification already seemingly having an impact on the camouflage of some fish^47^. It is possible that in industrially developing nations with high pollution levels, there are parallel examples to that of the peppered moth occurring at present. Advancing this understanding not only demonstrates evolution but illustrates the impact that humans can have on species interactions and fundamental biological processes, such as those between predators and prey.

## Methods

### Museum collection photography

Photographs of peppered moth (*Biston betularia*) specimens were required for digital image analysis. Photographic data were obtained from three museums in the south of England: Bristol Museum and Art Gallery, the Exeter Ark of the Royal Albert Memorial Museum and Art Gallery, and Oxford University Museum of Natural History. The Oxford collections are noteworthy as they contain the specimens collected by Kettlewell for his original bird predation and breeding experiments. Access was permitted to the specimen drawers for selection of the most intact *typica* and *carbonaria* forms of both sexes for sampling, with selection based on wing completeness. Due to the age and fragility of such specimens, care in handling the pinned insects was paramount.

We used a Nikon D7000 DSLR camera that had previously undergone a quartz conversion (Advanced Camera Services Limited, Norfolk, UK) by replacement of the internal UV filter with a transparent quartz sheet, as per previous studies^22,48^. This allowed for full spectral sensitivity throughout the avian-visible spectrum. A CoastalOpt 105 mm UV Macro APO interchangeable lens was attached whereby photographs in the human-visible spectrum were taken with a Baader UV-IR blocking filter (transmitting 400–700 nm) and ultraviolet (UV) photographs were taken using a Baader UV pass filter (transmitting 315–400 nm)^23^. All images were taken in RAW format. An area within the collections was selected with a minimal amount of natural and artificial light so not to interfere with the lighting equipment, which consisted of an Iwasaki EYE Colour Arc lamp connected to a Ventronic ballast, mounted onto a PhotxPro photographic lighting stand with an attached Elinchrom umbrella. The arc lamp was left to warm-up for 15 min to allow the bulb to reach at least 90% of its full light output. Prior to use, the UV blocking filter applied over the arc lamp was removed using a steel brush bit as to allow for a full spectrum of wavelengths. A cylindrical sheet of PTFE was placed around the bulb, or around the specimen(s) to be photographed if accessible, to evenly diffuse the light.

If appropriate, a moth was removed from the specimen drawer and pinned onto a neutral grey foam background. A maximum of three specimens per shot were photographed to ensure they were all uniform in size and wings were not out of the frame. To remove any effect of lighting variability, a light (95%) and dark (5%) reflectance standard (Labsphere, Congleton, UK) was included along with a scale bar positioned on the side closest to the specimens. The camera was positioned directly above using either a copy stand or a tripod with a flexible head, before being focused to obtain optimal sharpness. The exposure time was selected dependent on whether photographing with the visible or UV filter and optimised for correct exposure and to prevent saturation^49^. A camera shutter remote was used to minimise unnecessary noise or movement.

### Natural resting background camouflage analysis

Cryptic camouflage between the peppered moth and its natural resting backgrounds was explored by modelling avian vision to compare chromatic and achromatic contrasts. Woodlands in low-polluted areas were visited to obtain in situ photographs of plain tree bark and photos of bark possessing crustose lichen. Oak (*Quercus robar*) and ash (*Fraxinus excelsior*) trees were selected based upon their suitability as natural resting backgrounds as identified through previous peppered moth experiments^21,28^. Selection of crustose lichen species, *Lecidella elaeochroma* and *Graphis scripta*, as opposed to foliose lichen, was based upon previous work^4,21,45^, and because previous experiments show that crustose lichen is most commonly located on tree trunks and the lower sides of main branches^28^, which are the natural resting locations most frequented by the peppered moth^11^. Evidence against using foliose lichen is supported by ancestral populations found upon upper branches, whereas crustose lichens flourish underneath branches in unpolluted locations^4^. 130 individual trees were selected according to the previous outlined suitability, photographing 65 plain bark and 65 lichen covered bark specimens under neutral light during daylight hours, using the same Nikon D7000 camera and lenses as per museum specimen collection. A light and dark reflectance standard and scale bar was positioned in each photograph to correct scaling and any lighting differences encountered during image analysis.

To prepare the images for analysis, each photo was converted into a multispectral image using the open-access software ImageJ^50^ with the Image Calibration and Analysis Toolbox plugin^23^. A RAW image in both the UV and visible was selected for each specimen, and screened prior to this to check for overexposure. The light and dark reflectance standards were individually selected to normalise the channels and account for potential variation in light conditions^49^. Manual alignment was then conducted on the visible RGB and UV channels of the multispectral image 32-bit stack for accurate colour measurements. For each sample of the two different background types, a single *typica* or *carbonaria* moth morph was compared once by random selection, to determine how colour and luminance (lightness) contrasted between the moth and its background.

For modelling avian predator vision, the blue tit (*Cyanistes caeruleus*) was selected because previous studies have shown them to attack the peppered moth^10,11^, their foraging behaviour may be affected by UV cues^51^, and broadly their visual system is representative of many higher passerine birds^19,52^. We first used a well-established method of converting calibrated images from camera to animal colour space (predicted cone response data) using a polynomial mapping technique^23,49,53^ under D65 (daylight) lighting conditions. This method is highly accurate in generating cone response data compared to modelling using reflectance spectra^23,54,55^. Although we could have used a different irradiance spectrum (such as a green forest shade), this would not have affected the results for several reasons. First, the woodland is in fact not continuous shade but patches of trees and clearings, and thus a mixture of open skies and forest shade lighting. In addition, real moths and the models here would actually be exposed to a range of light conditions associated with time of day, depth in the forest/vegetation cover, and weather. In addition, like most other visual modelling, we account for the process of colour constancy, via the so-called von Kries transformation^56,57^, and as such, using a different irradiance spectrum has been shown to have little effect on predicted cone catch values^57,58^. Finally, to fully demonstrate that our modelling is robust for lighting, we compared the predicted cone catch values for 16 moths (eight *typica* and eight *carbonaria*) under both D65 and forest shade irradiance spectra. As expected, results were unchanged, with mean plus standard deviation cone catch values as follows: double (forest = 0.22 ± 0.13, D65 = 0.21 ± 0.13), longwave (forest = 0.27 ± 0.15, D65 = 0.27 ± 0.15), mediumwave (forest = 0.20 ± 0.13, D65 = 0.20 ± 0.13), shortwave (forest = 0.14 ± 0.10, D65 = 0.14 ± 0.10), ultraviolet (forest = 0.08 ± 0.06, D65 = 0.07 ± 0.06). In all cases, there was a very strong and significant correlation between the cone catch values under the two light conditions (Spearman’s rank correlation tests between forest and D65 for all receptors: *p* < 0.0001, *r* > 0.988).

Visual discrimination (level of camouflage) was assessed according to the widely used Vorobyev and Osorio receptor noise discrimination model^24^. On the basis of a recent detailed evaluation of estimates of receptor noise^59^, a Weber fraction of 0.1 was selected for colour discrimination with single cone (UV, shortwave, mediumwave, longwave) photoreceptor ratios of 1.00: 1.92: 2.68: 2.70, respectively^60^, while a Weber fraction of 0.2 was utilised for luminance. To quantify discrimination, JND values were calculated for both colour and achromatic contrast (luminance). JND calculations for the latter are based on double cones, and represent how discriminable two spectra are from one another under the assumption that visual discrimination is limited by receptor noise^24,61^. A JND value of 1.00 is taken as the discrimination threshold for birds, whereby JND < 1.00 indicates two objects cannot be distinguished even under optimal viewing conditions, and as values increase > 1.00 this denotes increasing contrast and greater differences in distinguishability^62^.

### Creation of artificial predation experiment targets

Artificial moth targets for the avian predation experiments followed a wide range of past experiments using similar targets based either on natural backgrounds or designed to mimic real species^25,26,29,63^. Targets were created using the multispectral images generated from the moth museum specimen photographs. As with a range of past work, images were converted to the predicted photon catches of the vision of the blue tit, under D65 lighting conditions, and then the output of a printer was calibrated in an iterative process^25,31,33^ such that the image colours (pixel values) converged to accurately match the real moths in terms of bird vision when reproduced. The match for every printed target was a JND value < 1.00, indicating indistinguishability from a real moth^25,63^. Generally, most previous work has tended to, for example, simply match the appearance of target wings to the average colour (in photon catches) of the substrate (e.g., tree bark^26,64^). Past work matching artificial prey items to Lepidopteran models has also tended to ensure that matches of colour are based on targets falling within the range of photon catch values of the real animal model^25^. Our work most closely follows other recent work on butterfly coloration^63,65^, which used a visual discrimination model to create matches to the real butterflies, with matches chosen when colours fell within 1–3 JNDs. Our approach is in fact even more detailed in that, unlike past studies, we did not create all targets per treatment as identical and simply matching an average model coloration, but instead we included individual variation by matching different individual targets to 100 unique individual moth models (see below). Therefore, our work is robust in accounting both for individual variation and in setting rigorous criteria, whereby models were only generated when colour and luminance matches to the real specimens were within 1.00 JND.

Once 50 individuals of each of the two morphs (*n* = 100) were correctly calibrated, they were scaled to ensure their size accurately matched real-life peppered moths; with each wing, on average, 221 mm^2^ in surface area. The specimens were printed using an HP LaserJet Enterprise 500 Colour M551 on Whatman Filter Paper No.1 12.5 cm stuck onto A4 plain paper. Using filter paper on which to print the targets was undertaken because this, unlike normal printer paper, reflects ultraviolet light and enabled us to match the UV reflectance of typical moths even though printer ink is incapable of printing UV itself^63^. Artificial wings were made waterproof using Plasti-Kote Matt Clear Acrylic spray paint, which permitted transmission / reflectance of UV.

An edible body was made of pastry, which has been shown in various previous experiments to be a safe, edible, and an effective measure of bird predation rates, including when attached to paper targets^27,66^. Following past protocols, lard and plain flour were mixed in a 1:3 ratio, before adding five drops of black food colouring for the *carbonaria* morphs, or a combination of approximately one red, one blue, three yellow and one black drop(s) for the typical morphs. White food colouring was used to add pattern to *typica* bodies. The pastry was rolled into 12 × 5 × 2 mm bodies, attached to the artificial wings using a 20-mm panel pin inserted into the centre of the pastry body, and left overnight in the freezer to set.

The creation of the edible bodies for the targets was somewhat constrained by both available food colourings and of aiming to match both colour and luminance simultaneously. We calculated, using the above methods and visual modelling, the colour and luminance contrasts of fifteen pastry bodies, of each morph, against the moths themselves and natural resting backgrounds. For luminance, results for bodies to moths were: *carbonaria* (mean JND plus standard error: 3.838 ± 0.733) and *typica* (3.809 ± 0.731). For colour, matches were: *carbonaria* (16.106 ± 1.477) and *typica* (4.991 ± 0.641). The comparisons of bodies to respective natural backgrounds for luminance were: *carbonaria* (3.773 ± 0.348) and *typica* (1.660 ± 0.309). For colour, matches were: *carbonaria* (13.721 ± 1.328) and *typica* (2.783 ± 0.148). Therefore, results demonstrate relatively low distinguishability of bodies to both moths and backgrounds, with the exception of *carbonaria* for colour. However, this difference is highly unlikely to explain our survival results for multiple reasons (see Discussion).

### Artificial predation experiments

Artificial moth targets modelled to avian visual systems were used as a proxy for peppered moths to determine morph survival rate through a field predation experiment. This approach is well tested and closely follows a range of past work^25–27,29–34,63–65^. Data collection was conducted in June 2017 to coincide with peppered moth emergence between May and August 2017. Ten locations within mixed deciduous woodlands in low-polluted areas over the south of England were selected based upon availability of crustose lichen. In Cornwall, these were Argal Reservoir, Penryn (50°9′11ʺ N, 5°8′11ʺ W), Lady’s Wood, Truro (50°17′24.2ʺ N, 5°3′42.2ʺ W), St Clement Wood, Truro (50°17′29.2ʺ N, 5°3′16.1ʺ W), Kennall Vale, Ponsanooth (50°11′42.3ʺ N, 5°8′59.1ʺ W), and Devichoys Wood, Truro (50°15′17.5ʺ N, 5°3′23.6ʺ W). In Hampshire, Holybourne Down, Alton (51°10′46.6ʺ N, 0°56′52.3ʺ W) and Chawton Park Wood, Alton (51°7′31.1ʺ N, 1°1′52.4ʺ W) were visited. The experiment followed a randomised block design^26^ of 500 samples over ten blocks along a non-linear transect ranging between 1–1.5 km in length. In each block were 25 replicates of each treatment – 50 targets in total. These were pinned at a height of 1.7 m on a singular lichen-possessing tree at least 10 m apart from one another. If a tree did not have crustose lichen at this height, a further 1 m was walked along the transect until a suitable tree was found, and then the next subsequent tree would be 10 m apart. Moths were haphazardly selected from a bag, to avoid selection bias, and pinned onto trunks and under branches because these areas were shown to be the natural resting locations most frequented by the moth^11^. The targets were pinned at orientations within 20° of the horizontal plane as this is naturally exhibited behaviour in geometrid moths^41^ (Fig. 3). Additionally, targets were pinned into position at the centre of the lichen patch being sampled. While there may be a risk of unconscious bias with this and various previous similar experiments, we believe the procedures here make that unlikely. Each transect was checked at three time intervals: 6, 24, and 48 h. At each interval, all moths were visited to determine whether any predation, or another outcome, had occurred. Results were censored whereby 1 represented bird predation or censored 0 if the artificial moth had survived to 48 h, was missing, or another incident had occurred such as predation from a slug (identified by slime trails^26^). Bird predation was evident if the artificial target had rips from >50% of the body^19,46^ and/or the wings were wholly or partially removed. As outlined in past studies^64^, the process of recording predation events with such a large number of targets is impractical, and indeed experiments like ours here have been validated on multiple occasions. Other studies on artificial prey have also deduced avian predation from marks made on clay bodies^63^. The woodland used in this experiment is comparable to that of other studies, which included information on species present and observed as taking prey items, including: blue tits (*Cyanistes caeruleus*), great tits (*Parus major*), European robins (*Erithacus rubecula*), chaffinches (*Fringilla coelebs*), blackbirds (*Turdus merula*), and house sparrows (*Passer domesticus*)^32,64^. Furthermore, direct observations of birds attacking live and pinned peppered moths have been made in past studies^6,11^.

### Statistical analysis

Image analysis statistics were undertaken using the open-access software R (v.3.4.0)^67^. A generalised linear model was performed on both colour and luminance JNDs, with background type as the response variable and morph the explanatory variable. Model residuals were checked for assumptions of homogeneity of variance and a normal distribution, for which colour JND data for both bark and lichen required a log transformation to correct for skewness. For the field experiment, a Nonparametric Distribution Analysis (Right Censoring) was performed using Minitab Statistical Software 18^68^ with Kaplan–Meier estimation to measure survival probabilities, along with survival curves comparison using the log-rank test^69,70^ on the factor treatment. A nonparametric analysis was performed because survival data frequently do not follow a pre-specified given distribution. All significance levels were set at *α* = 0.05.

### Data availability

The data reported in this paper are available in Supplementary Data 1.

## Electronic supplementary material

Description of Additional Supplementary Files

Supplementary Data 1
