# Supplementary material for: Avian vision models and field experiments determine the survival value of peppered moth camouflage
Source: Commun Biol. 2018 Aug 17;1:118. doi: 10.1038/s42003-018-0126-3 (PMC6123793; doi:10.1038/s42003-018-0126-3)
Supplement: Supplementary file 1 — Description of Additional Supplementary Files [file 42003_2018_126_MOESM1_ESM.pdf]

Description of Additional Supplementary Files

File Name: Supplementary Data 1

Description: Modelled avian cone catch response data corresponding to the typical and melanic museum moth specimens and of the plain and lichen tree backgrounds, for the UV, shortwave, mediumwave, longwave, and double cones.

Results of visual discrimination models (JNDs) of moths versus each background type for colour and luminance.

Results of visual discrimination models (JNDs) of artificial target bodies used in the survival experiments versus each background type and the wing target backgrounds for colour and luminance.

Modelled avian cone catch response data corresponding to typical and melanic museum moth specimens, for the UV, shortwave, mediumwave, longwave, and double cones modelled under both forest shade and D65 irradiance spectra.

Results of the artificial field predation experiments on melanic and typical targets over time for each experimental block.
